# Supplementary material for: UK Healthcare Workers’ Experiences of Major System Change in Elective Surgery During the COVID-19 Pandemic: Reflections on Rapid Service Adaptation
Source: Int J Health Policy Manag. 2021 Sep 1;11(10):2072–82. doi: 10.34172/ijhpm.2021.101 (PMC9808275; doi:10.34172/ijhpm.2021.101)
Supplement: Supplementary file 1 — Topic Guide. [file ijhpm-11-2072-s001.pdf]

Article title: UK Healthcare Workers' Experiences of Major System Change in Elective Surgery During the COVID-19 Pandemic: Reflections on Rapid Service Adaptation

Journal name: International Journal of Health Policy and Management (IJHPM)

Authors' information: Georgina Singleton[1,2]¶, Anna Dowrick[3]¶*, Louisa Manby4, Harrison Fillmore[5], Aron Syverson[4], Sasha Lewis-Jackson[1,2], Inayah Uddin[1,2], Kirsi Sumray4, Elysse Bautista-González[4,2], Ginger Johnson[1,2], Cecilia Vindrola-Padros[1,2]

[1]Department of Targeted Intervention, University College London, London, UK.

[2]Rapid Research Evaluation and Appraisal Lab (RREAL), University College London, London, UK.

[3]Nuffield Department of Primary Care Health Sciences, University of Oxford, Oxford, UK.

[4]Institute of Epidemiology and Healthcare, University College London, London, UK.

[5]Department of Anthropology, University College London, London, UK.

¶ Both authors contributed equally to this paper.

(*Corresponding authors: anna.dowrick@phc.ox.ac.uk)

Supplementary file 1. Topic guide

1. **Background: Can you tell me about your role?**
   - *Can you tell me a bit about your role? (e.g. Daily tasks, department, responsibilities)*

2. **Have you been in contact with patients who had suspected and/or confirmed COVID-19?**
   - *In what capacity?*
   - *How have you found working around these patients?*
   - *PPE physical effects? (E.g. dehydration, discomfort, restriction in movement, difficulties communicating)*
   - *How has PPE impacted the type of care you provide patients?*
   - *What psychological/emotional impact did this have on you?*

3. **Could you briefly explain how Covid-19 has impacted on elective and emergency surgery at your health facility?**
    - *How has this affected your normal daily tasks/responsibilities? Change of role?*
    - *What tasks are you able to do more or less effectively?*
    - *Has there been an impact on staff's ability to make diagnoses and act on them?*
    - *Has the supply of drugs, equipment and PPE been affected?*
    - *Have staff been redeployed from or within your health facility*

4. **What do you think about the initial decision to cancel or delay non-emergency surgeries?**
    - *Was it made at the right time?*
    - *How was the decision communicated to you?*
    - *Did you express any concerns about the strategy and if so, were you listened to?*
    - *Have any decisions during Covid-19 have been clinically-led by yourself or colleagues at your facility? Which?/Why not?*
    - *What effect did this decision to delay/cancel surgeries have on care-seeking by patients?*
    - *How do you think the delay or cancellation of surgeries will have impacted on patient outcomes and mental health?*
    - *Have you felt that delaying or cancelling patients' surgeries has had an effect on your own mental health? In what way?*

5. **What were the preparedness strategies implemented locally (department, hospital or Trust)?**
    - *Did you feel these strategies were enough?*
    - *What do you feel was particularly successful?*
    - *Should the Trust have prepared differently?*
    - *Did you receive any training? (including but not limited to PPE training such as mental health and well-being training)*
    - *Did you have access to guidance on PPE?*

6. **NHS England issued a six-week action plan to restart non-urgent Covid-19 care.**

- *Are you aware of the procedure for how and when services will restart at your health facility?*
- *What impact will this have on your role and your daily tasks?*
- *Are staff being given the opportunity to provide feedback and through what means?*
- *What do you think about the handling and coordination of this pandemic with regards to surgery? What was done well and what would have been improved?*

7. **Do you currently have any concerns or fears in relation to ...**
    - *Restarting elective surgeries (timing, staffing, equipment, PPE, capacity, patient safety)*
    - *Other work concerns (response efforts, PPE, services)*
    - *The national effort*

8. **Over the past months, have you experienced any problems with aspects of your daily life such as sleeping, eating, concentration, or additional worries or anxiety?**

9. **Mental health support (to address risk of moral injury, trauma and developing severe mental health problems)**
    - *Are you aware of any support available for staff wellbeing and mental health?*
    - *Have you had the opportunity to talk about your mental health with your supervisor/team leader?*
    - *Have you had worrying experiences in the last week? Did you receive support after? If so, what type of support? (including formal and informal support)*
    - *Interactions between peers: Do you have time to socialise with your team? What has changed with COVID-19?*

10. **Is there anything else you would like to mention that you feel is important?**
